# Conditional cause-specific survival after chemotherapy and local treatment for primary stage IV breast cancer: A population-based study

**DOI:** 10.3389/fonc.2022.800813

**Published:** 2022-08-05

**Authors:** Min Xiao, Pin Zhang

**Affiliations:** ^1^ Department of Medical Oncology, National Cancer Center/ Hospital, Chinese Academy of Medical Sciences and Peking Union Medical College, Beijing, China; ^2^ Department of Intensive Care Unit, Fujian Medical University Cancer Hospital, Fujian Cancer Hospital, Fuzhou, China

**Keywords:** Breast cancer, conditional survival, prognosis, therapy, SEER program

## Abstract

**Background:**

Conditional survival (CS) represents the probability of surviving for additional years after the patient has survived for several years, dynamically describing the survival rate of the patient with the varying time of survival. The aim of this study was to evaluate the conditional cause-specific survival (CCSS) after chemotherapy and local treatment for metastatic breast cancer, and to identify the prognostic factors affecting the CCSS.

**Methods:**

Patients diagnosed with primary stage IV breast cancer in the Surveillance, Epidemiology, and End Results (SEER) database from 2010 to 2015 were included. CS is defined as the probability of additional survival for *y* years after the patient had survived *x* years with the calculation formula CCSS (*x* | *y*) = CSS (*x* + *y*)/CSS (*x*), where CSS(*x*) indicates the patient’s cause-specific survival rate at the time of *x* years. Cox proportional hazard models were used to evaluate predictors of CCSS.

**Results:**

A total of 3,194 patients were included. The 5-year CSS was 39%, whereas the 5-year CCSS increased to 46%, 57%, 71%, and 85% after the diagnosis of 1, 2, 3, and 4 years. For patients with adverse clinical pathological features, CCSS had more pronounced increase with survival time and is more different from the CSS at diagnosis. No matter at the time of diagnosis or 1 year or 3 years after diagnosis, HER2 status, local treatment, and multisite metastasis were independent prognostic factors that affect the long-term survival of patients (all *P* < 0.05).

**Conclusion:**

The 5-year CCSS of patients with stage IV breast cancer was extended as the survival years increased. HER2 status, multisite metastasis, and local treatment were independent prognostic factors even 3 years after diagnosis.

## Introduction

Breast cancer is the most common malignant tumor and is also the most frequent cause of death from cancer in women (1). Globally, over two million patients are diagnosed annually and over 600,000 die from the disease (2). About 5–10% patients at diagnosis have metastases (3). Despite the use of various traditional systemic treatments such as chemotherapy, endocrine treatment, and targeted therapy, the overall survival (OS) of patients with metastatic breast cancer is still not so satisfactory. Recently, some studies showed that chemotherapy combined with local treatment including primary tumor site surgery or radiotherapy or both may improve the prognosis of advanced breast cancer (4–6).

Most survival rates reported in the literature are static, being calculated from the day of diagnosis or surgery (7–10). This statistical method could only reflect the continuous hazard ratio and survival rate of patients from the beginning of follow-up. Since the survival rate, death risk, and risk ratio of patients will change with the extension of survival time, this approach has limitations, especially for long-term survival. Conditional survival (CS) represents the probability of surviving a certain number of years after diagnosis treatment based on the time the patient has already survived (11). Compared with the traditional survival evaluation, CS can provide more accurate information for long-term prognosis and is more meaningful in the process of follow-up. Thus, it has been used in many kinds of malignant tumors, such as gastrointestinal, liver, pancreatic, and urinary tract cancer (12–15).

As we know, there is no report on the conditional cause-specific survival (CCSS) in patients with metastatic breast cancer who underwent chemotherapy combined with local treatment. Our study aims to evaluate the dynamic cause-specific survival (CSS) of this type of population and prognostic factors that change with time.

## Material and methods

### Data source and study population

**Figure 1:**
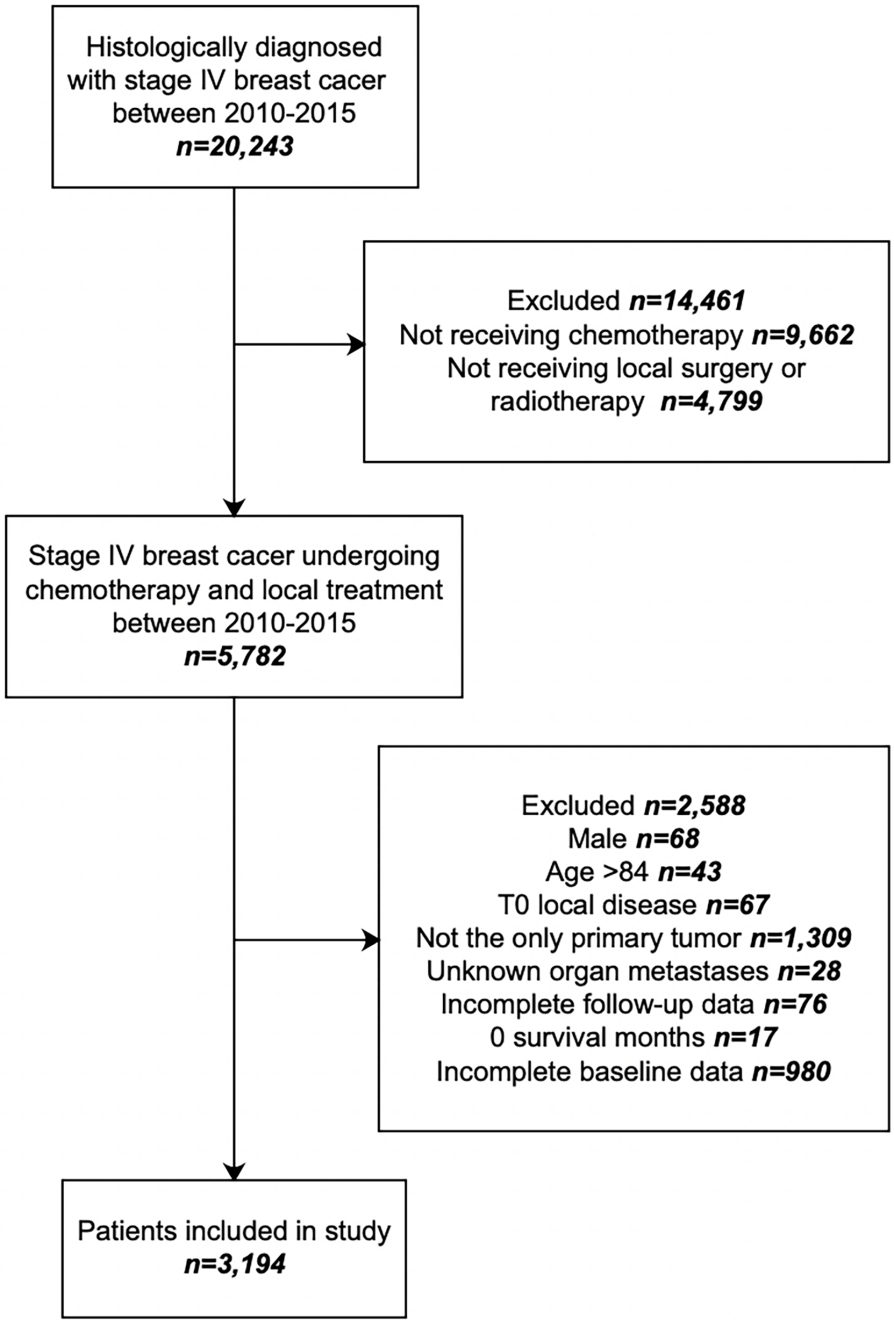
Study flow chart.

A retrospective cohort study was performed with data extracted from the Surveillance, Epidemiology, and End Results (SEER) database. The SEER program collects and publishes cancer incidence and survival data from population-based cancer registries covering approximately 34.6% of the U.S. population. The inclusion criteria were as follows: (1) patients histologically diagnosed as stage IV breast cancer according to the 7th edition of the American Joint Committee on Cancer (AJCC) TNM classification between 2010 and 2015, and (2) chemotherapy combined with local surgery and/or radiotherapy were performed. The exclusion criteria were as follows: (1) male, (2) more than 84 years old, (3) T0 local disease, (4) not the only primary tumor, (5) lack of information on distant metastatic lesion, (6) incomplete follow-up data, (7) 0 survival month, and (8) incomplete baseline data. A total of 3,194 cases entered the final analysis (**Figure 1**). All data obtained included age at diagnosis, race, tumor grade, human epidermal growth factor receptor 2 (HER2), estrogen receptor (ER), progesterone receptor (PR) status, AJCC TNM stage, metastatic organ, treatment, and follow-up information. The SEER program identifies only the first course of therapy, defined as those recorded in the treatment plan at diagnosis and administered before disease progression or recurrence. Surgery in the current research refers to the primary lesion (16). SEER data are publicly available, and a signed Research Data Agreement form was required to access the database. No institutional review board approval was required for this study.

### Statistical analysis

CSS was measured by the time between diagnosis and breast cancer–related death. Survival curves were constructed according to the Kaplan–Meier (K–M) method, and difference curves were analyzed using the log-rank test.

CS is defined as the possibility of surviving an additional number of *y* years given that a patient has already survived for *x* years. The CCSS formula is CCSS (*x* | *y*) = CSS (*x* + *y*)/CSS (*x*), where CSS (*x*) represents the cause-specific survival at *x* year calculated by the K–M curve. For example, CS for surviving another year among patients who had already survived 4 years, CCSS (1|4), was calculated by dividing the 5-year K–M survival estimate CSS (5) by the 4-year survival estimate CSS (4).

Multivariate Cox proportional-hazards regression was performed to evaluate the hazard of CSS at the time of diagnosis and CCSS for multiple survival periods (1 and 3 years after diagnosis). For instance, to compute the CCSS at 1 year after diagnosis, 1-year survivors were selected. After subtraction of 12 months from their survival time, a multivariate analysis was performed. Only the variables that were prognostic with *P*-value less than 0.1 in the analysis of the previous period were selected and incorporated in the next period’s multivariate analysis sequentially. Differences were statistically significant when *P* < 0.05. Statistical analyses were performed using the SPSS 22.0 statistical software.

## Results

### Clinicopathological characteristics

This study included 3,194 breast cancer patients who met the criteria in the SEER database (**Table 1**). Most of the patients were younger than 65 years old (78.4%). Majority of the patients were White (71.1%) followed by Black (19.6%). The most frequent histopathological grade was poorly differentiated (60.7%). Bone metastasis (35.6%) was the most common site of metastasis, followed by lung metastasis (10.9%) and brain metastasis (1.6%). In terms of treatment, more than 70% of the patients received chemotherapy combined with surgery, of which 1,222 (38.3%) patients received chemotherapy combined with surgery and radiotherapy.

**Table 1:** Baseline and treatment characteristics.

	No. of patients (%, n=3194)
Age	
<65 years	2503 (78.4)
≥65 years	691 (21.6)
Race	
White	2270 (71.1)
Black	625 (19.6)
Other	299 (9.3)
AJCC 7th, T Stage	
T1	326 (10.2)
T2	1135 (35.5)
T3	590 (18.5)
T4	1143 (35.8)
AJCC 7th, N Stage	
N0	487 (15.2)
N1	1369 (42.9)
N2	572 (17.9)
N3	766 (24.0)
Grade	
Well	145 (4.5)
Moderate	1078 (33.8)
Poorly	1939 (60.7)
Anaplastic	32 (1.0)
HER2 Status	
Negative	2079 (65.1)
Positive	1115 (34.9)
Breast type	
HR+/HER2+	690 (21.6)
HR+/HER2-	1415 (44.3)
HR-/HER2+	425 (13.3)
HR-/HER2-	664 (20.8)
ER Status	
Negative	1148 (35.9)
Positive	2046 (64.1)
PR Status	
Negative	1594 (49.9)
Positive	1600 (50.1)
Metastatic organ	
Bone	1136 (35.6)
Brain	52 (1.6)
Liver	281 (8.8)
Lung	349 (10.9)
Multisites	843 (26.4)
Other	533 (16.7)
Treatment	
Chemo+radio	792 (24.8)
Chemo+surgery	1180 (36.9)
Chemo+surgery+radio	1222 (38.3)

AJCC, the American Joint Committee on Cancer; ER, Estrogen receptor; PR, Progesterone receptor; HER2, Human epidermal growth factor receptor 2; chemo, chemotherapy; radio, radiotherapy.

### Comparison of CSS and CCSS

**Figure 2:**
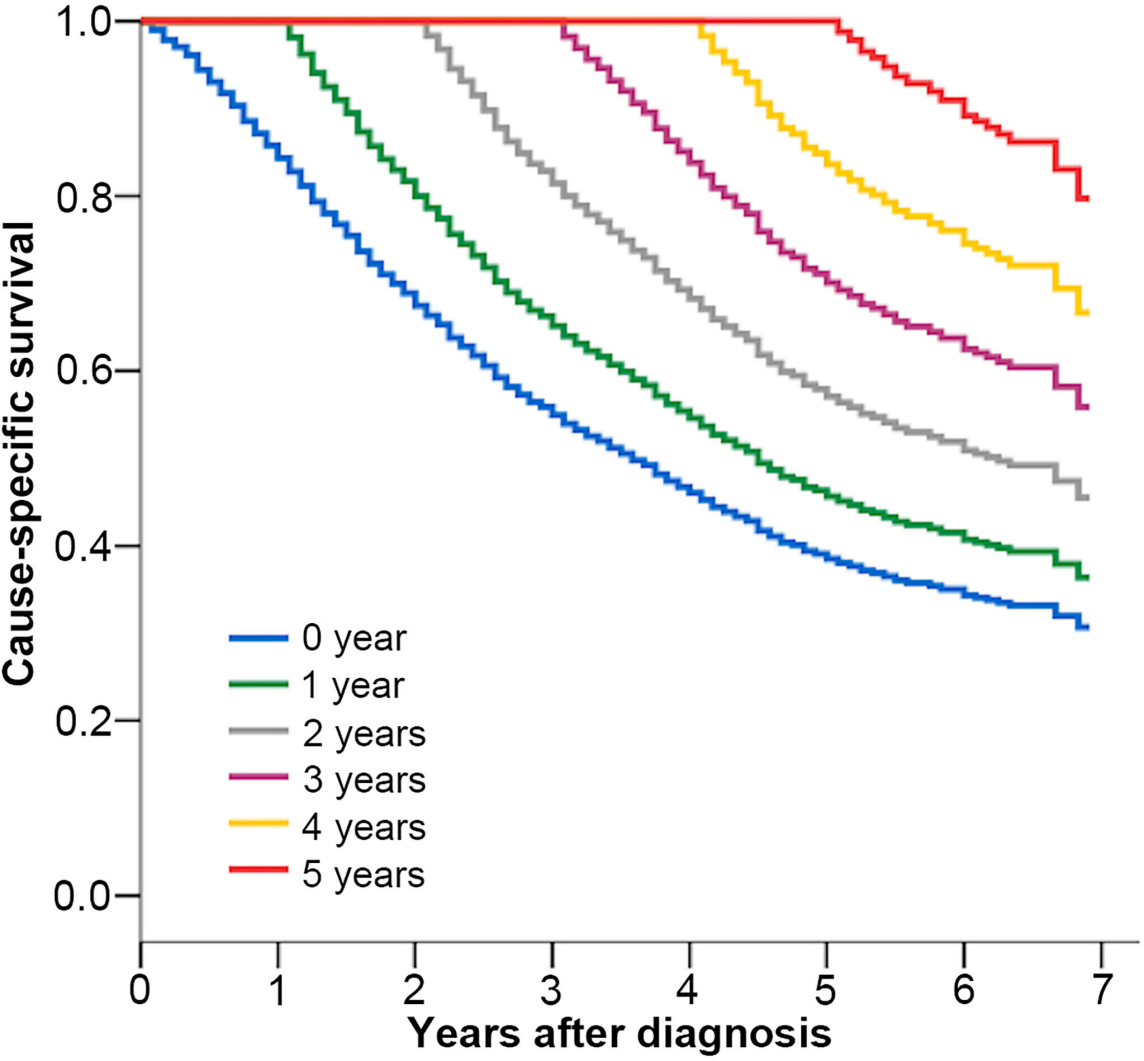
Kaplan–Meier estimates of cancer-specific survival after diagnosis (0 year) and conditional cancer-specific survival, according to years already survived after diagnosis (1–5 years).

With a median follow-up time of 26 (1–83) months until 2018, the CSS of patients at 1, 3, and 5 years was 84%, 55%, and 39%, respectively. The CCSS related to the number of years are shown in **Table 2**, and the K–M survival curves are shown in **Figure 2**. The 5-year CCSS increased from 39% directly after diagnosis to 46% (Δ 7%), 57% (Δ 18%), 71% (Δ 32%), and 85% (Δ 46%), given 1, 2, 3, and 4 years already survived, respectively. The longer the patients have survived, the more likely they are to survive for additional years. This growth leveled off after many years.

**Table 2:** Conditional cause-specific survival estimates.

Total years of survival after diagnosis	Probability of survival (%)					
	Years already survived by patient					
	0	1	2	3	4	5
1	84					
2	68	81				
3	55	65	81			
4	46	55	68	84		
5	39	46	57	71	85	
6	34	40	50	62	74	87

The probability of survival after diagnosis is shown in relation to the number of years already survived. For example, if a patient has survived 2 years after diagnosis, the probability of achieving 3-year survival after diagnosis is 81 percent and of achieving 5-year survival after diagnosis is 57 percent.

### Factors associated with CSS and CCSS rates

Multivariate analysis showed that age, race, AJCC T, N categories, tumor grade, HER2, ER, PR status, metastatic organ, and treatment were independent prognostic factors for CSS of metastatic breast cancer (all *P* < 0.05, **Table 3**) at diagnosis. For patients surviving for 1 year after diagnosis, multivariate analysis identified that T4, poorly grade, HER2 positive, brain, and multisite metastasis were independent risk factors (all *P* < 0.05), whereas ER positive, PR positive, and surgery or surgery combined with radiotherapy were independent protective factors (all *P* < 0.05). After 3 years of diagnosis, only HER2 positive (HR = 0.598, *P* < 0.001), multisite metastasis (HR = 1.621, *P* = 0.002), and surgery (HR = 0.507, *P* < 0.001) or surgery combined with radiotherapy (HR = 0.521, *P* < 0.001) were still independent prognostic factors.

**Table 3:** Multivariable Cox proportional hazards analysis of risk factors associated with cause-specific survival.

	At diagnosis (n=3194)		1 year after diagnosis (n=2585)		3 years after diagnosis (n=1071)	
	Hazard ratio	P	Hazard ratio	P	Hazard ratio	P
Age		0.001		0.542		NA
<65 years	Reference		Reference			
≥65 years	1.223 (1.082-1.381)	0.001	1.050 (0.898-1.228)	0.542		
Race		0.041		0.253		NA
White	Reference		Reference			
Black	1.102 (0.973-1.248)	0.127	1.029 (0.880-1.203)	0.723		
Other	0.846 (0.702-1.021)	0.082	0.839 (0.671-1.049)	0.123		
AJCC 7th, T Stage		0.002		0.011		0.132
T1	Reference		Reference		Reference	
T2	1.136 (0.934-1.382)	0.202	1.219 (0.960-1.548)	0.105	1.475 (0.926-2.351)	0.102
T3	1.091 (0.882-1.349)	0.442	1.146 (0.884-1.486)	0.303	1.668 (1.014-2.742)	0.044
T4	1.342 (1.104-1.632)	0.003	1.415 (1.112-1.800)	0.005	1.724 (1.077-2.761)	0.023
AJCC 7th, N Stage		0.085		0.147		NA
N0	Reference		Reference			
N1	0.937 (0.807-1.089)	0.396	0.992 (0.823-1.196)	0.934		
N2	0.995 (0.833-1.1887)	0.952	1.038 (0.835-1.289)	0.739		
N3	1.113 (0.942-1.314)	0.209	1.189 (0.967-1.461)	0.100		
Grade		<0.001		<0.001		0.610
Well	Reference		Reference		Reference	
Moderate	1.462 (1.085-1.970)	0.013	1.322 (0.948-1.844)	0.100	1.470 (0.838-2.577)	0.179
Poorly	1.952 (1.453-2.622)	<0.001	1.711 (1.230-2.381)	0.001	1.431 (0.811-2.523)	0.216
Anaplastic	1.847 (1.100-3.103)	0.020	1.517 (0.804-2.862)	0.198	1.334 (0.376-4.730)	0.655
HER2 Status		<0.001		<0.001		<0.001
Negative	Reference		Reference		Reference	
Positive	0.386 (0.342-0.435)	<0.001	0.380 (0.330-0.439)	<0.001	0.598 (0.457-0.783)	<0.001
ER Status		<0.001		0.001		0.566
Negative	Reference		Reference		Reference	
Positive	0.674 (0.585-0.777)	<0.001	0.739 (0.621-0.880)	0.001	0.893 (0.608-1.312)	0.566
PR Status		<0.001		<0.001		0.271
Negative	Reference		Reference		Reference	
Positive	0.525 (0.456-0.606)	<0.001	0.497 (0.420-0.588)	<0.001	0.829 (0.594-1.157)	0.271
Metastatic organ		<0.001		<0.001		<0.001
Bone	Reference		Reference		Reference	
Brain	3.295 (2.392-4.538)	<0.001	2.367 (1.445-3.877)	0.001	1.470 (0.360-6.002)	0.591
Liver	1.332 (1.083-1.639)	0.007	1.261 (0.986-1.611)	0.064	0.783 (0.459-1.336)	0.369
Lung	1.065 (0.884-1.282)	0.508	1.128 (0.908-1.401)	0.277	0.936 (0.598-1.464)	0.771
Multisites	1.877 (1.639-2.149)	<0.001	1.645 (1.394-1.942)	<0.001	1.621 (1.190-2.208)	0.002
Other	0.846 (0.712-1.005)	0.057	0.805 (0.660-0.983)	0.033	0.677 (0.455-0.978)	0.038
Treatment		<0.001		<0.001		<0.001
Chemo+radio	Reference		Reference		Reference	
Chemo+surgery	0.503 (0.439-0.576)	<0.001	0.559 (0.468-0.667)	<0.001	0.507 (0.357-0.720)	<0.001
Chemo+surgery+radio	0.392 (0.341-0.450)	<0.001	0.494 (0.415-0.588)	<0.001	0.521 (0.372-0.729)	<0.001

AJCC, the American Joint Committee on Cancer; ER, Estrogen receptor; PR, Progesterone receptor; HER2, Human epidermal growth factor receptor 2; chemo, chemotherapy; radio, radiotherapy, NA, Not Available.

### Subgroup analysis of CSS and CCSS rates

**Figure 3:**
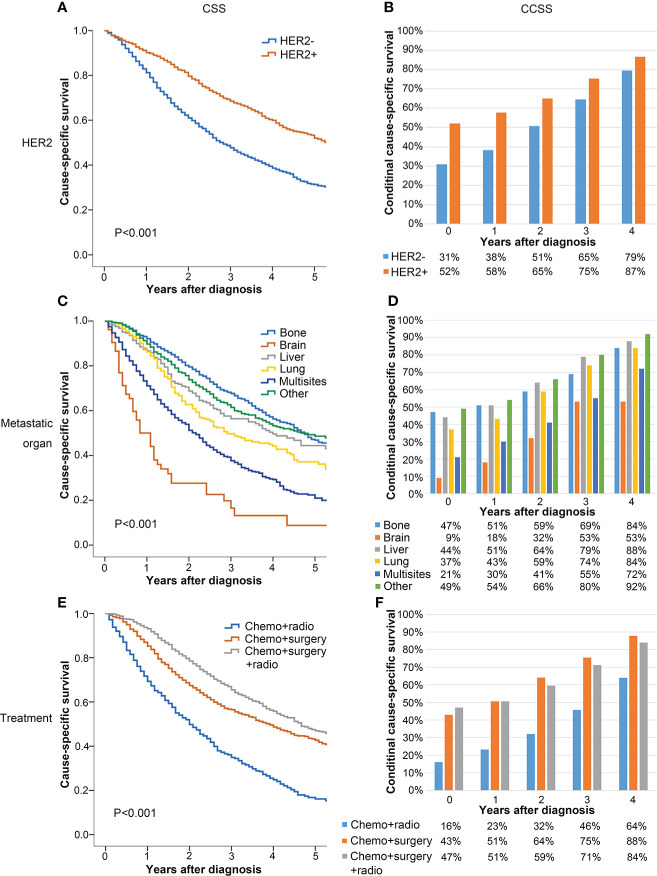
Comparison between CSS **(A, C, E)** and CCSS **(B, D, F)** according to HER2 **(A, B)**, metastatic organ **(C, D)**, and treatment **(E, F)**.

All patients were divided into subgroups according to the independent prognostic factors to evaluate their effects on CSS and CCSS. **Figure 3** shows that the 5-year CSS of HER2 positive patients was significantly better than that of HER2 negative patients (52% vs. 31%, *P* < 0.001, **Figure 3A**). In the subgroup analysis according to the metastatic site, the 5-year CSS of patients with brain metastasis (9%) was significantly worse than that of patients with bone (47%), liver (44%), and other sites (49%) (*P* < 0.001, **Figure 3C**). The 5-year CCSS of patients with bone, liver, and other site metastasis who have survived 4 years after diagnosis increased to 84%, 88%, and 92%, respectively, whereas the 5-year CCSS of patients with brain metastasis was only 53% (**Figure 3D**), which indicated that patients with brain metastasis disease at diagnosis still experience disease progression despite surviving 4 years. The subgroup analysis according to the treatment methods showed that the prognosis of patients who underwent surgery with or without radiotherapy was significantly better than that of patients who only underwent radiotherapy (5-year CSS of radiotherapy = 16%, 5-year CSS of surgery = 43%, 5-year CSS of surgery combined with radiotherapy = 47%, *P* < 0.001) (**Figure 3E**).

In each subgroup, CSS showed a downward trend, whereas the 5-year CCSS gradually went up with the passage of survival time. In each subgroup, the 5-year CCSS was better than the 5-year CSS. Moreover, the difference between the CSS and the 5-year CCSS was more significant in patients with poor clinicopathological factors at baseline. In contrast, this difference was relatively small in patients with good initial clinicopathological factors at baseline. For example, the 5-year CSS (baseline) of patients with HER2 positive was 52%, whereas the 5-year CCSS of 4 years after diagnosis was 87% (Δ 35%). For patients with HER2 negative, the 5-year CCSS was 31% at diagnosis and the 5-year CCSS increased to 79% (Δ 47%) at 4 years after diagnosis (**Figure 3B**).

## Discussion

To the best of our knowledge, this is the first study evaluating the CS of metastatic breast cancer. More than 3,000 cases of metastatic breast cancer with chemotherapy and local treatment in the SEER database were included in this study. It has been found that although the population has poor prognosis with the 5-year CSS only 39%, the 5-year CCSS increased with the extension of survival time. For patients who have survived for 4 years, the 5-year CCSS is as high as 85%, especially for patients with adverse prognostic factors. Furthermore, HER2 status, multisite metastasis, and treatment were independent prognostic factors at the time of diagnosis, and their prognostic effects persisted until 3 years after diagnosis.

CS represents the possibility that a patient can survive a certain number of years after diagnosis or treatment based on the time the patient has already survived. It can dynamically describe the survival rate of patients as time progresses (17). In this study, the 5-year CCSS of metastatic breast cancer increased year by year with the increase in survival years. For example, the probability of survival at 5 years after diagnosis went from 39% at 0 years to 71% at 3 years. In the subgroup analysis, this increasing trend was more obvious in patients with poor clinicopathological factors. The prognosis of surviving patients with high risk factors will be close to those of patients with some low risk factors as time goes on, which can reduce anxiety and improve the quality of life, especially for high-risk patients. For instance, the 5-year CSS of HER2 positive and HER2 negative patients at diagnosis were 52% and 31% (difference of 21%), and the 5-year CCSS of 4 years after diagnosis were 87% and 79% (difference of 8%). This may be due to the rapid death of high-risk patients after diagnosis. In the traditional survival analysis, patients with risk factors tend to have worse CSS. Therefore, cumulative survival analysis is somewhat crude for accurately assessing long-term survival, especially for patients who have survived for a period of time (18).

Currently, the treatment of metastatic breast cancer is still controversial. Three prospective randomized trials (the MF07-01, an Indian study, and the recent ECOG-ACRIN 2108 Trial) have shown different effects of the local treatments (19–22). The 3-year OS was similar between systemic therapy and primary surgery arms in all of them. However, the MF07-01 trial showed a better 5-year and 10-year OS in patients who underwent local treatment followed by system therapy compared with those who received only system therapy. There are some pitfalls in the above studies. The imbalance of baseline variables, insufficiency of system therapy, and high tumor burden are thought to lead to bias. Thus, it is very difficult to conduct a perfect random trial about the local therapy for primary stage IV breast cancer in a real world. Of particular note is oligometastatic disease, which can achieve long-term remission and even be cured through different treatment strategies (23). The BOMET MF14-01 study showed that bone metastasis only (especially oligometastatic bone and solitary bone) may take more advantage from local surgery (24). The subgroup analysis of the MF07-01 trial also favored the fact that the solitary bone metastasis was the proper candidate for local therapy (19, 20). So, it is very important to recognize those patients who can really benefit from the local treatment, and CS may be a better predictor of continued survival for people with long-term survival benefits.

Our study found that surgery combined with radiotherapy as the local treatment was more efficient compared with surgery or radiotherapy alone. The 5-year CSS increased from 16% to 43% (Δ 27%, *P* < 0.001), and it further increased to 47% (Δ 31%, *P* < 0.001) in patients accepting surgery combined with radiotherapy. Lian et al. collected data from SEER between 2004 and 2012 and also drew a similar conclusion (25). The 3-year CSS were 35.9%, 57.1%, and 63.9% in patients who underwent radiotherapy alone, surgery alone, and surgery combined with radiotherapy. Our study suggests that the local treatment can affect the prognosis for a long time. Due to the inability to obtain metastatic tumor load from the SEER database, we were unable to perform further analysis. In addition, the patients who have a good initial prognosis (low tumor burden, metastatic clearance with system therapy, fewer complications, and younger age), as evaluated subjectively by the physician, were more likely to opt for surgery, leading to bias.

Previous studies have shown that age, HER2 status, hormone receptor state, metastatic sites, and treatment were important factors affecting the prognosis of metastatic breast cancer (8, 26, 27), but there is no study on the prognostic factors for patients with metastatic breast cancer who have survived for several years. In this study, we found that age, race, grade, HER2, ER, PR status, metastatic organ, and local treatment were independent prognostic factors for CSS, which is consistent with the previous studies (8). However, at 1 year and 3 years after diagnosis, only HER2 status, metastatic organ, and local treatment continued to affect the prognosis. With HER2-targeted therapy, the prognosis of HER2 positive metastatic breast cancer has been improved (28). Our study also showed that the prognosis of HER2 positive patients was significantly better than HER2 negative, and this factor continued to influence the long-term survival during follow-up, which verified that the targeted treatment of HER2 had long-term survival benefits to the metastatic breast cancer. The common metastatic sites were bone, lung, brain, and liver, of which the prognosis of brain and multisite metastasis was the worst (29, 30). In this study, the 5-year CSS of brain metastasis and multisite metastasis patients were only 9% and 21%, and the latter remained an independent risk factor for prognosis as years of survival increased. Obviously, the more the metastases, the higher the tumor burden. As a result, these patients have a poor prognosis.

This study has some limitations. First of all, this is a retrospective study and inevitably leads to selection bias. Second, information such as the treatment of targeted and endocrine, the sequence of chemotherapy and surgery, and the therapeutic effect evaluation cannot be obtained from the SEER database. However, this is the first study to assess the 5-year CCSS of metastatic breast cancer and to analyze the potential factors that continue to influence the prognosis. The results of this study can be used as an important basis for improving treatment options as well as the prognosis of patients with metastatic breast cancer in the future.

## Conclusions

CCSS of metastatic breast cancer was dynamic and increases with each additional year survived. Compared with CSS, CCSS provided a more individualized prognosis. Furthermore, HER2 status, multisite metastasis, and local treatment were independent prognostic factors that continued to influence the survival of metastatic breast cancer. These patients seemed to benefit more from surgery combined with radiotherapy.

## Data availability statement

The original contributions presented in the study are included in the article/Supplementary Material. Further inquiries can be directed to the corresponding author.

## Author contributions

PZ and MX designed and performed the research. MX performed the statistical analyses, interpreted the data, and wrote the manuscript. Both authors critically reviewed and approved the manuscript.

## Conflict of interest

The authors declare that the research was conducted in the absence of any commercial or financial relationships that could be construed as a potential conflict of interest.

## Publisher’s note

All claims expressed in this article are solely those of the authors and do not necessarily represent those of their affiliated organizations, or those of the publisher, the editors and the reviewers. Any product that may be evaluated in this article, or claim that may be made by its manufacturer, is not guaranteed or endorsed by the publisher.
